# Coenzyme Q10 and Obesity: An Overview

**DOI:** 10.3390/antiox14070871

**Published:** 2025-07-16

**Authors:** David Mantle, Sofia Kozhevnikova, Steen Larsen

**Affiliations:** 1 Pharma Nord (UK) Ltd., Telford Court, Morpeth, Northumberland NE61 2DB, UK; skozhevnikova@pharmanord.com; 2 Department of Biomedical Sciences, Faculty of Health Sciences, University of Copenhagen, DK-2200 Copenhagen, Denmark; 3 Clinical Research Centre, Medical University of Bialystok, 15-089 Białystok, Poland

**Keywords:** obesity, mitochondrial dysfunction, oxidative stress, ferroptosis, inflammation, coenzyme Q10

## Abstract

There is evidence for the involvement of mitochondrial dysfunction, oxidative stress, ferroptosis, and inflammation in the pathogenesis of obesity. This, in turn, indicates a novel potential therapeutic role for supplemental coenzyme Q10 (CoQ10) in the management of obesity, due to the role of CoQ10 in promoting normal mitochondrial function, as an antioxidant, and as an anti-ferroptotic and anti-inflammatory agent. In the present article we have, therefore, reviewed the potential role of CoQ10 in the prevention and treatment of obesity. A potential role for supplementary CoQ10 (in combination with selenium) in preserving skeletal muscle mass in obese individuals undergoing weight loss procedures is also discussed.

## 1. Introduction

Obesity refers to the accumulation of excess body fat that presents a risk to health, and it is a growing global health concern, affecting millions worldwide and placing significant burdens on healthcare systems [1]. Individuals are classified as obese when their body mass index (BMI; the weight divided by the square of the height in meters) is over 30 kg/m^2^. A BMI value in the range 25–30 is classified as being overweight. Although widely used, BMI as a measure of obesity has its limitations, since it does not differentiate between fat and muscle mass, or consider body fat distribution, which can result in a misleading interpretation of an individual’s health risks.

**Figure 1:**
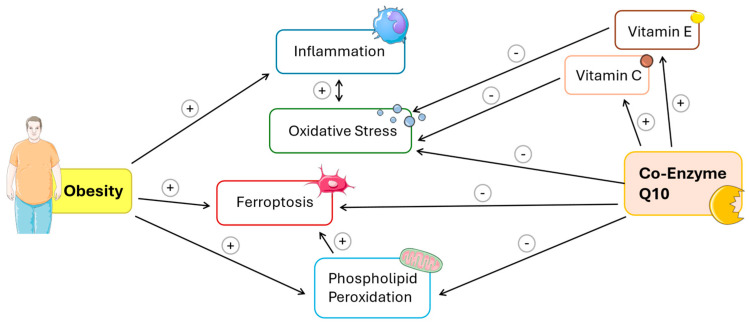
Schematic representation of the mechanistic interplay between obesity and mitochondrial dysfunction, and the regulatory actions of Coenzyme Q10 (CoQ10), based on current literature. Arrows are annotated with “+” (promoting effect) or “−” (inhibitory effect) to indicate the directionality of influence. Obesity exerts a multifaceted impact on cellular homeostasis. It promotes inflammation (+), oxidative stress (+), ferroptosis (+), and phospholipid peroxidation (+). Inflammation and oxidative stress are closely interlinked: inflammation increases oxidative stress (+), and, in turn, oxidative stress can sustain or amplify inflammatory responses (+), forming a reinforcing loop. Ferroptosis, a regulated form of cell death driven by iron-dependent lipid peroxidation, is independently promoted by both obesity (+) and increased phospholipid peroxidation (+). Coenzyme Q10 (Co-Q10), a lipid-soluble antioxidant and essential component of the mitochondrial electron transport chain, attenuates several key pathological features. It reduces inflammation (−), oxidative stress (−), ferroptosis (−), and phospholipid peroxidation (−), suggesting a broad protective role across these interconnected pathways. Additionally, Co-Q10 supports the action of vitamins E and C (+), which themselves exert anti-inflammatory (−) and antioxidant (−) effects.

In the UK, the current prevalence of obesity is approximately 25% of the adult population. Causes of obesity include excessive dietary calorie intake, lack of exercise, medical conditions such as an under-active thyroid, the effect of certain medications such as steroids, and genetic factors [2]. Obesity increases the risk of developing a number of disorders, including metabolic syndrome/type II diabetes, non-alcoholic fatty liver disease (NAFLD), cardiovascular disease (heart disease, hypertension, atherosclerosis, stroke), certain types of cancer (endometrial, breast, colon), and osteoarthritis [3]. The treatment of obesity in the first instance is based on dietary modification and exercise; other therapeutic options include the use of drugs such as orlistat, GLP-1 receptor agonists (GLP-1 RAs) such as semaglutide, and bariatric (weight loss) surgery [4].At the biochemical level, there is evidence for the involvement of mitochondrial dysfunction, oxidative stress, and inflammation in the pathogenesis of obesity, as reviewed in the following sections. This, in turn, indicates a novel potential therapeutic role for supplemental coenzyme Q10 (CoQ10) in the management of obesity, due to the role of CoQ10 in promoting normal mitochondrial function, as an antioxidant, and as an anti-inflammatory and anti-ferroptotic agent [5]. As the prevalence of obesity continues to rise, exploring innovative therapeutic approaches such as supplementation with CoQ10 could provide new avenues for intervention. In the present article, we have, therefore, reviewed the potential role of CoQ10 in the prevention and treatment of obesity. The inter-relationship between obesity, mitochondrial dysfunction, inflammation, and ferroptosis has been summarised in Figure 1.

## 2. Mitochondrial Dysfunction and Obesity

Mitochondria have a key role in the metabolism of normal adipocytes, oxidizing metabolic fuels to produce ATP and generating heat during thermogenesis. However, mitochondrial function is impaired in obese individuals. The supply of excess nutrients associated with obesity can saturate the tricarboxylic acid cycle and the electron transport chain [6], reducing the ability to regenerate oxidized nicotinamide adenine dinucleotide (NAD+). The resulting mitochondrial dysfunction is characterised by a reduction in mitochondrial mass, impaired mitochondrial membrane potential, reduced mitochondrial oxygen respiration, decreased fatty acid oxidation, increased mitochondrial Ca^2+^ flux, and low cellular ATP levels [7]. This, in turn, can lead to excessive production of free radical species and associated oxidative stress, which, in turn, exacerbates inflammation, as reviewed in the following sections of this article. These processes have been described in obesity as occurring mainly in peripheral tissues. However, some studies have already shown that obesity is also associated with changes in the central nervous system, with alterations in the blood–brain barrier (BBB) and in cerebral structures, such as the hypothalamus and hippocampus.

Within adipose tissue, obesity can induce mitochondrial dysfunction in cell types other than adipocytes. During obesity, the progressive accumulation of lipids in adipocytes results in hypertrophy and hyperplasia of adipose tissue, leading to the infiltration of macrophages. The macrophages, in turn, absorb lipids released from adipocytes. When the accumulation of lipids within macrophages becomes excessive, then mitochondrial dysfunction results [8,9].

Mitochondrial dysfunction is characteristic of both rodent and human obesity. Thus, reduced levels of mitochondrial amount or function (biogenesis and oxidative phosphorylation) have been described in adipose tissues of obese human subjects; downregulated mitochondrial DNA, altered mitochondrial oxidative function/respiratory chain Complex I-IV activities, reduced fatty acid oxidation, and depleted ATP levels have been observed in muscle, as well as adipose tissue from obese compared to healthy weight individuals [10,11,12,13,14,15,16,17].

The above studies have focused on obesity-related mitochondrial dysfunction in adipose tissue, but other tissues are also affected by obesity and could potentially benefit from improved mitochondrial function; examples include obesity-related mitochondrial dysfunction/oxidative stress/inflammation in brain tissue [18], in skeletal muscle tissue [19], and in renal tissue [20].

In animal models, a number of studies have highlighted a reduction in mitochondrial mass and function in adipose tissues of obese ob/ob and db/db mice [21,22]. Xia et al. [23] demonstrated that high-fat diet feeding causes mitochondrial fragmentation and reduced oxidative capacity in inguinal white adipocytes from male mice. In aged mice, Li et al. [24] found a reduction specifically in mitochondrial complex IV activity, resulting in reduced fatty acid oxidation and subsequent adipocyte hypertrophy. The latter study is of note in that aging, per se, is known to affect mitochondrial function [25], and mitochondrial dysfunction in obese, aged subjects may represent a novel therapeutic target in the otherwise difficult-to-treat disorder of sarcopenic obesity [26].

Recent research has focused on the role of the mitochondrial unfolded protein response (UPRmt) in obesity; the UPRmt is a protective mechanism that maintains mitochondrial function during stress by activating genes that produce chaperones and proteases to repair or remove damaged proteins [27]. Whilst initially beneficial in restoring mitochondrial health, prolonged UPRmt activation in obesity can contribute to metabolic imbalances and worsen dysfunction. Recent findings highlight that obesity-induced metabolic stress activates the UPRmt, a dual-effect quality-control pathway that influences mitochondrial function and metabolic balance depending on the tissue, cell type, and physiological state [28,29,30]. Since UPRmt is activated by mitochondrial dysfunction to restore homeostasis, the authors speculate that CoQ10 supplementation could modulate UPRmt activity by enhancing mitochondrial efficiency and reducing the accumulation of misfolded proteins [31]. However, further targeted research is necessary to elucidate the specific effects of CoQ10 on UPRmt activation and its implications for conditions like obesity. The potential role of mitochondrial transplantation to replace defective mitochondria in obesity has also been the subject of recent research [32].

With regard to mitochondrial dysfunction, as noted in the introduction, CoQ10 has a key role in the normal functioning of mitochondria. CoQ10 has a key role as an electron carrier (from complex I and II to complex III) in the mitochondrial electron transport chain during oxidative phosphorylation. CoQ10 is also a key component in the reactions mediated by other mitochondrial enzymes; for example, it is also involved in the metabolism of pyrimidines, fatty acids, and mitochondrial uncoupling proteins, as well as in the regulation of the mitochondrial permeability transition pore [33,34]. The potential role of supplementary CoQ10 in the management of obesity-related mitochondrial dysfunction is addressed in a subsequent section of this article.

## 3. Oxidative Stress and Obesity

Cells constantly generate reactive oxygen species (ROS) from multiple sources, with mitochondria being the primary site of production. Within mitochondria, electron leakage from the electron transport chain (ETC) to combine with oxygen leads to the formation of superoxide radicals [35]. Additionally, uncoupling proteins can alter mitochondrial ROS levels by allowing protons to re-enter the mitochondrial matrix, reducing the proton gradient and decreasing superoxide formation. Beyond mitochondria, enzymes, such as NAD(P)H oxidase, xanthine oxidase, lipoxygenase, and cyclooxygenase, also contribute to ROS production [36].

While often associated with oxidative stress, ROS plays a crucial role in cellular energy regulation. When fuel supply exceeds demand, excess NADH drives mitochondrial ROS production, signaling a surplus and triggering insulin secretion to promote fuel storage. As energy reserves are balanced, ROS levels naturally decline. Conversely, when fuel is scarce, NADH and ROS levels drop, reflecting the need for energy intake [37]. Mitochondrial ROS removal depends on NADPH, produced through glucose and fat metabolism, positioning redox reactants as key regulators of metabolic balance [38]. To prevent oxidative damage, cells maintain a robust defence system of antioxidants, including specialised enzymes, vitamins, and CoQ10, ensuring that ROS functions as precise metabolic signals rather than sources of harm [39].

Although ROS plays an important role in normal cellular signaling, increased levels or prolonged exposure to ROS can lead to pathological changes to a variety of cellular components, including the modification of DNA, RNA, carbohydrates, proteins, and lipids. An imbalance between ROS-generating and antioxidant protective systems is known as oxidative stress. Malondialdehyde, F-2 isoprostanes, 8-iso prostaglandin F2α, and protein carbonylation are established biomarkers used to quantify oxidative stress in plasma or other tissues [40].

As noted in the previous section of this article, obesity-induced mitochondrial dysfunction results in increased ROS production. In addition, the activity of antioxidant enzymes such as superoxide dismutase and glutathione peroxidase has been reported to be significantly reduced in obese individuals [41,42]; the levels of other antioxidants, including vitamin C and vitamin E, are also decreased in obesity [43,44]. The combination of increased ROS generation and reduced antioxidant levels results in oxidative stress characteristic of obesity. Numerous studies have reported elevated levels of oxidative stress biomarkers in obese individuals, including malondialdehyde, F-2 isoprostanes, and oxidized low-density lipoprotein [45,46]. Oxidative stress not only damages cellular components but also activates redox-sensitive transcription factors such as nuclear factor-κB (NF-κB) and activator protein-1 (AP-1), triggering the release of pro-inflammatory cytokines such as TNF-α and IL-6. This sustained inflammatory response contributes to the chronic low-grade inflammation characteristic of obesity [47], further exacerbating metabolic dysfunction, as discussed in the following section.

CoQ10 (particularly in its reduced ubiquinol form) serves as an important lipid-soluble antioxidant protecting cellular membranes, both mitochondrial and extra-mitochondrial (Golgi apparatus, lysosomes, endoplasmic reticulum, peroxisomes) from free radical-induced oxidative stress. In addition to acting as an antioxidant directly, CoQ10 is also involved in the regeneration of the antioxidants vitamin C and vitamin E, respectively [33].

## 4. Apoptosis, Ferroptosis, and Obesity

Apoptosis is a tightly regulated process of programmed cell death, which can occur as a result of mitochondrial dysfunction associated with obesity; pro-apoptotic factors, such as cytochrome c, are released from damaged mitochondria into the cytoplasm to induce apoptosis mediated by caspase-type proteolytic enzymes [48]. Adipocyte apoptosis has been reported in both obese mice and obese humans [49]. A considerable number of preclinical studies have been reported in which the administration of CoQ10 has inhibited apoptosis; for example, after spinal cord injury in rats [50] and in a mouse cell model of diabetes [51]. Supplementation with CoQ10 has been shown to increase levels of the anti-apoptotic protein B-cell lymphoma-2 (Bcl-2) in obese rats [52].

Another consequence of mitochondrial dysfunction associated with obesity is ferroptosis. Ferroptosis is an iron-dependent form of cell death characterized by iron accumulation and extensive lipid peroxidation; it differs morphologically, genetically, and biochemically from other cell death types, including apoptosis. The CoQ10–NAD(P)H/ferroptosis suppressor protein (SP1) pathway is responsible for inhibiting phospholipid peroxidation and ferroptosis. FSP1 is a membrane-associated oxidoreductase that catalyses the transport of reduced NADH analogs of CoQ10 into the lipid bilayer, thereby inhibiting lipid peroxidation [53]. Several preclinical studies have demonstrated the action of CoQ10 or its structural analogues in inhibiting ferroptosis; these include models of epilepsy [54], subarachnoid haemorrhage [55], myocardial infarction [56], Parkinson’s disease [57], and acute liver injury [58].

## 5. Inflammation and Obesity

Mitochondrial dysfunction in adipocytes results in reduced synthesis of adiponectin, the most abundant adipokine synthesized by adipocytes, which has systemic anti-inflammatory action [59]. In addition to mitochondrial dysfunction and oxidative stress in adipocytes as an inducer of inflammation in obesity, as discussed in the previous sections of this article, similar dysfunctions in macrophages are also responsible for obesity-related inflammation. During obesity, the progressive accumulation of lipids in adipocytes leads to hypertrophy and hyperplasia of adipose tissue, resulting in the infiltration of macrophages.

In normal individuals, macrophages comprise approximately 5% of the total cells in adipose tissue. However, in obese individuals, this increases to approximately 50%. Mitochondria are essential for the normal functioning of macrophages. Excessive lipid accumulation in macrophages results in obesity-induced mitochondrial dysfunction and subsequent oxidative stress, which drives the activation of the NLRP3 inflammasome and the release of pro-inflammatory cytokines such as interleukin-1β (IL-1β) and tumour necrosis factor alpha (TNF-alpha) [60,61]. This inflammatory response, in turn, results in the decreased sensitivity of insulin target cells characteristic of obese individuals [8].

CoQ10 performs a number of cellular functions of potential relevance to the immune system. Firstly, the immune response has intensive energy requirements, and an adequate supply of CoQ10 is, therefore, required to enable the various cell types of the immune system to function optimally. Secondly, since phagocytic cells destroy invading pathogens via the production of free radicals, the antioxidant action of CoQ10 may protect phagocytic cells from self-destruction caused by their generation of free radicals. Thirdly, CoQ10 is able to directly modulate the action of genes involved in inflammation and may have a role in controlling the release of pro-inflammatory cytokines [62].

## 6. CoQ10 and Obesity

In preclinical studies, reduced levels of CoQ10 in subcutaneous adipose tissue were identified in obese ob/ob mice by Bour et al. [63]; the same authors identified a similar deficiency of CoQ10 in adipose tissue of obese human subjects. Goncalves et al. [64] showed that in obesity, hepatic ubiquinone synthesis is impaired, causing an increase in the ubiquinol/ubiquinone ratio, resulting in excessive ROS production via reverse electron transport from Complex I.

Supplementation with CoQ10 reduced elevated plasma lipid profiles and decreased mRNA expression of the pro-inflammatory cytokine TNF-α in adipose tissues of ob/ob mice [65]. In the latter study, decreased mRNA expression of the lipogenic enzymes fatty acid synthase and acetyl-CoA carboxylase 1, and the glycerogenic enzyme phosphoenolpyruvate carboxykinase was responsible for the lipid-lowering effect of CoQ10.

In C57BL/6 mice with diet-induced obesity, CoQ10 supplementation reduced the levels of oxidative stress and inflammation in hepatic tissue [66]. In KKAy obese mice, supplementation with the ubiquinol form of CoQ10 enhanced mitochondrial function, improved lipid metabolism, and ameliorated obesity by reducing white adipose tissue content [67]. In C57BL/6 mice with diet-induced obesity, supplementation with CoQ10 improved mitochondrial function and oocyte competence [68]. Fink et al. [69] demonstrated that supplementation with the CoQ10 analogue mitoquinone reduced fat mass and oxidative stress in C57BL/6 mice with diet-induced obesity. Supplementary CoQ10 improved lipid metabolism and reduced fat mass in rats with diet-induced obesity [70]. Preclinical studies supplementing CoQ10 in animal models of obesity have been summarised in Table 1.

In clinical studies, Grenier-Larouche et al. [71] measured the CoQ10 content and redox state in omental and subcutaneous adipose tissue depots of lean, overweight, and obese women; obese women had reduced CoQ10 levels and increased lipid peroxidation levels. Mehmetoglu et al. [72] reported reduced lipid-adjusted CoQ10 levels in sera from obese individuals. Proteomic analysis of visceral adipose tissue from obese individuals showed reduced levels of a number of mitochondrial proteins, including quinone biosynthesis protein COQ9 [73].

Not all clinical studies have reported beneficial outcomes following CoQ10 supplementation in obesity. Thus, a randomised controlled trial by Lee et al. [74] found CoQ10 supplementation had no significant effect on the levels of serum lipid profiles or oxidative and inflammatory biomarkers. However, this outcome may have been influenced by the mean BMI of the obese (27.9 ± 2.3 kg/m^2^) subjects. A meta-analysis of randomised controlled trials by Ghavami et al. [75] failed to demonstrate any significant effect of supplementary CoQ10 on anthropometric indices (body weight, body mass index, waist circumference). However, this study had a number of limitations; for example, included trials were performed in subjects with a wide range of different medical disorders making the interpretation of results difficult. In addition, included trials ignored the measurement of the baseline levels of CoQ10 in study participants, so the lack of efficacy could be related to low levels of CoQ10 in participants.

Beneficial effects of supplementary CoQ10 have been reported in obese patients with disorders such as polycystic ovary syndrome [76] or type II diabetes [77]. However, such studies have, in general, been excluded from the present review because of the confounding effect of the comorbidities.

Obesity is linked to alterations in cardiolipin, a phospholipid located in the inner mitochondrial membrane, with a key role in maintaining normal mitochondrial function. These alterations to cardiolipin include a reduction in cardiolipin levels, as well as structural changes to the phospholipid’s acyl chains, in turn promoting mitochondrial dysfunction and increased oxidative stress [78]. In rats that were fed an obesogenic diet, supplementation with the CoQ10 analogue mitoquinone (MitoQ) improved mitochondrial function and reduced oxidative stress in liver tissue by increasing the levels of cardiolipin [79]. However, it should be noted that a beneficial effect following mitoquinone administration was observed in the process of inducing obesity, not in the obese animals. In addition, it only preserved mitochondrial function. However, it did not ameliorate liver lipid contents. This suggests that CoQ10 does not directly improve obesity-associated MASLD itself; it may only function to maintain mitochondrial function by supplementing CoQ10, which is lost due to the obesity- or lipid-induced oxidative stress.

## 7. Weight Loss and CoQ10/Selenium

In the previous section of this article, the role of CoQ10 deficiency and supplementation with regard to obesity was discussed. However, CoQ10 is also of relevance for obese individuals wishing to lose weight. Skeletal muscle mass is important for overall health. Muscle mass loss is normally seen with aging [80]. During weight loss, lean body mass loss is also seen, with a magnitude of around 25–39% of the weight lost [81]. It seems as if the loss in muscle mass does not affect muscle strength [82], but strength is only one function of the muscle. Muscle mass also has many important metabolic roles (myokine production, amino acid reserve, glycaemic regulation, and immune function). Therefore, it is highly relevant to focus on how to preserve muscle mass during weight loss.

It has been reported that combining exercise (resistance or endurance) with a weight-loss intervention preserves muscle mass [83]. Testosterone, growth hormone (GH), and Insulin-like growth factor-1 (IGF-1) all seem to be important for muscle mass growth. It has been reported that GH and IGF-1 are decreased in obese participants compared to lean control participants, and that a bariatric surgery-induced weight loss increases IGF-1 and GH [84]. Interestingly, it has also been reported that IGF-1 increases with a diet-induced weight loss in obese participants, but when participants were followed for a longer time, the levels were comparable to before weight loss [85]. It has previously been shown that supplementation with CoQ10 and selenium increases IGF-1 in a group of older participants [86]. Cavedon and colleagues [87] investigated the effect of selenium supplementation on body composition in participants with obesity after a mild hypocaloric diet. They supplemented obese participants with 240 µg of selenium per day for 3 months and measured body composition before and after the intervention. Participants in both the selenium and placebo group lost approximately 3–4 kg during the 3 months, but the selenium group gained muscle mass and lost a considerable amount of fat [87]. Unfortunately, the authors did not measure selenium concentration in that study. The studies outlined above raise the question of whether it would be beneficial to supplement participants with CoQ10, and especially selenium, when losing weight (diet or medical) in order to try to maintain more muscle mass. This is especially interesting at the moment when many different weight loss drugs are on the market, and many people are using them.

## 8. Discussion and Conclusions

1. In the present article, we have reviewed evidence for the involvement of mitochondrial dysfunction, oxidative stress, ferroptosis, and inflammation in the pathogenesis of obesity. Due to the key role of CoQ10 in promoting normal mitochondrial function, as an antioxidant, as an anti-ferroptosis agent, and as an anti-inflammatory agent [34,88], there is a rationale for investigating the potential role of supplementary CoQ10 in the management of obesity.

2. There is evidence for reduced levels of CoQ10 in adipose tissue or serum/plasma in both animal models of obesity and in obese human subjects. A number of preclinical studies have reported beneficial effects of CoQ10 supplementation in animal models of obesity, including improved lipid metabolism and reduced levels of oxidative stress and inflammatory biomarkers. However, to date, there have been no randomised controlled clinical trials supplementing CoQ10 in obese individuals without confounding comorbidities, and this remains an area for future research.

3. While this review has focused on mitochondrial dysfunction (and associated oxidative stress/inflammation) in adipose tissue, many other tissues (e.g., brain, skeletal muscle, kidney) are also affected by obesity and could potentially benefit from improved mitochondrial function. No relevant studies were identified in which supplementary CoQ10 had been administered to address obesity-related mitochondrial dysfunction in such tissues, and this remains a further area for future research.

4. Obesity is associated with lysosomal dysfunction, in turn resulting from an obesity-linked increase in oxidative stress, causing ROS-induced damage to lysosomal membranes [89]. CoQ10 has a key role in mediating normal lysosomal function. In addition to its role in maintaining the lysosomal acidic pH, it protects lysosomal membranes from ROS-induced oxidative damage [90]. The potential role of supplemental CoQ10 in preventing obesity related lysosomal dysfunction is another area of research requiring further investigation.

5. In addition to its potential role in the management of obesity-related mitochondrial dysfunction, a possible role for supplementary CoQ10 in the preservation of skeletal muscle mass in obese individuals undergoing weight loss procedures has been identified by the authors.

6. Obesity is an independent risk factor for the development of heart failure, in turn, involving mitochondrial dysfunction and increased oxidative stress [91,92]. This, therefore, suggests a potential role for supplementary CoQ10 in the treatment of heart failure in obese individuals. No randomised controlled clinical trials were identified to date in which supplementary CoQ10 was used to treat heart failure in obese individuals without significant comorbidities, and this remains another area for future research. In this regard, it is of note that in the Q-SYMBIO randomised controlled trial, in which individuals with heart failure were supplemented with 300 mg CoQ10 per day for 2 years, cardiac-related mortality was reduced by 50% [93].

7. As noted in the Introduction section of this article, in addition to heart failure, obesity increases the risk of developing a number of co-morbidities, including NAFLD [94]. Two randomized controlled clinical trials have been conducted to date, supplementing CoQ10 in NAFLD patients. In both cases, 100 mg/day of CoQ10 supplemented for 4 weeks [95] or 12 weeks [96], respectively, resulted in significant reductions in blood markers for inflammation and liver damage. Similarly, randomised controlled trials have demonstrated the benefit of supplementary CoQ10 in improving glycaemic control in patients with type II diabetes [97], and in improving renal function in patients with chronic kidney disease [98], both disorders being co-morbidities of obesity [99,100].

8. This article has focused on the consequences of mitochondrial dysfunction in obesity and the potential beneficial effects of supplementation with CoQ10. However, there is a rationale in general terms to investigate the potential benefit of supplementation of CoQ10 in combination with other metabolites of relevance to promoting normal mitochondrial function, including B-vitamins, L-carnitine, and alpha lipoic acid [101]. Some clinical studies based on this approach have already been reported with some benefit in obese individuals. For example, a randomised controlled clinical trial supplementing a number of nutrients designed to improve mitochondrial function, including CoQ10 and alpha-lipoic acid, reported significant improvements in weight, body composition, and metabolic biomarkers in obese subjects [102]. In this regard, it is of note that randomised controlled clinical trials supplementing individual supplements as above have reported benefit in obese subjects. Thus, supplementation with alpha lipoic acid (600 mg/day for 24 weeks) resulted in weight loss and improved oxidative stress and inflammation [103]. Supplementation with the NAD precursor beta-nicotinamide mononucleotide reduced body weight and improved cholesterol parameters [104]. Several meta-analyses of randomised controlled trials supplementing L-carnitine in obese subjects concluded this nutrient was effective in reducing weight, BMI, or waist circumference [105,106,107]. Supplementation with multiple nutrients of relevance to normal mitochondrial function in obese subjects is, therefore, another area requiring further research.

9. As noted previously in this article, there is evidence for reduced levels of CoQ10 in obesity. The question arises whether the reduction in CoQ10 levels might result from an adverse effect of obesity on CoQ10 biosynthesis. The biosynthesis of CoQ10 occurs via a complex pathway involving at least 10 steps, each of which is mediated via a specific enzyme [108]. No studies were identified in which the effect of obesity on these various biosynthetic steps had been investigated, and this remains yet another area of research open to further investigation.

In conclusion, most of the evidence relating to CoQ10 and obesity is derived from animal model studies, with relatively few clinical studies identified relating to CoQ10 supplementation in obese individuals without serious confounding comorbidities. We have, therefore, suggested a number of areas for further research in human subjects as described in items 2 to 9 above.

## Figures and Tables

**Table 1:** Summary of preclinical studies supplementing CoQ10 in animal models of obesity.

Study	Model System	CoQ10 Dosage	Outcome
Carmona et al. [65]	ob/ob mice	10 mg/kg/day CoQ10 injected ip for 13 days.	Reduction in elevated plasma lipid profiles and decreased mRNA expression of the pro-inflammatory cytokine TNF-α in adipose tissue.
Sohet et al. [66]	C57BL/6 mice	1% CoQ10 in feed for 8 weeks.	Reduced levels of oxidative stress and inflammation in hepatic tissue.
Xu et al. [67]	KKAy mice	0.3% CoQ10 in feed for 12 weeks.	Enhanced mitochondrial function, improved lipid metabolism, and ameliorated obesity via reduction of white adipose tissue content.
Boots et al. [68]	C57BL/6 mice	22 mg/kg CoQ10 three times weekly via subcutaneous injection for 6 weeks.	Improved mitochondrial function and oocyte competence.
Al-Ghamdi et al. [70]	Rats (high fat diet)	10 mcg CoQ10/kg/day via intragastric tube for 6 weeks.	Improved lipid metabolism and reduced fat mass.

## Data Availability

Not applicable.
